# Global Image Properties Predict Ratings of Affective Pictures

**DOI:** 10.3389/fpsyg.2020.00953

**Published:** 2020-05-12

**Authors:** Christoph Redies, Maria Grebenkina, Mahdi Mohseni, Ali Kaduhm, Christian Dobel

**Affiliations:** ^1^Experimental Aesthetics Group, Institute of Anatomy I, Jena University Hospital, Friedrich Schiller University, Jena, Germany; ^2^Department of Otolaryngology and Institute of Phonatry and Pedaudiology, Jena University Hospital, Friedrich Schiller University, Jena, Germany

**Keywords:** experimental aesthetics, affective pictures, image properties, emotion, subjective ratings

## Abstract

Affective pictures are widely used in studies of human emotions. The objects or scenes shown in affective pictures play a pivotal role in eliciting particular emotions. However, affective processing can also be mediated by low-level perceptual features, such as local brightness contrast, color or the spatial frequency profile. In the present study, we asked whether image properties that reflect global image structure and image composition affect the rating of affective pictures. We focused on 13 global image properties that were previously associated with the esthetic evaluation of visual stimuli, and determined their predictive power for the ratings of five affective picture datasets (IAPS, GAPED, NAPS, DIRTI, and OASIS). First, we used an SVM-RBF classifier to predict high and low ratings for valence and arousal, respectively, and achieved a classification accuracy of 58–76% in this binary decision task. Second, a multiple linear regression analysis revealed that the individual image properties account for between 6 and 20% of the variance in the subjective ratings for valence and arousal. The predictive power of the image properties varies for the different datasets and type of ratings. Ratings tend to share similar sets of predictors if they correlate positively with each other. In conclusion, we obtained evidence from non-linear and linear analyses that affective pictures evoke emotions not only by *what* they show, but they also differ by *how* they show it. Whether the human visual system actually uses these perceptive cues for emotional processing remains to be investigated.

## Introduction

Affective pictures have become increasingly popular in psychological, neuroscientific and clinical research on emotions over the last two decades (Horvat, 2017). According to the Web of Science database, the number of articles that cite publications retrieved under the topic “affective picture” rose from 34 articles in the year 2000 to 3,590 articles in the year 2018. Researchers have studied the role of affective pictures in cognitive and physiological processes such as fluency, autonomic arousal, pupil size and facial expression (Bernat et al., 2006; Bradley et al., 2008; Albrecht and Carbon, 2014; Lang et al., 1993; Lench et al., 2011; Snowden et al., 2016). Their effect on neurophysiological processes has been investigated using event-related potentials (Junghöfer et al., 2001; Olofsson et al., 2008; Weinberg and Hajcak, 2010) and fMRI (Satpute et al., 2015). In group studies, emotional pictures have been used, for example, to study gender differences (Sabatinelli et al., 2004; Lithari et al., 2010), mental illness (Shapira et al., 2003) and child development (McManis et al., 2001).

Visual material is particularly effective in eliciting emotions in human observers (Lench et al., 2011). It is generally believed that pictorial content plays a decisive role in evoking emotions when humans view affective images (Weinberg and Hajcak, 2010). However, physical image properties that represent low-level perceptual features can also have an effect on emotional processing (Delplanque et al., 2007; Satpute et al., 2015). The human visual system processes low-level features fast and automatically, allowing humans to recognize not only the general meaning of scenes (Oliva and Torralba, 2006) at a glance (“gist perception,” Bachmann and Vipper, 1983), but also to evaluate affective aspects of images, such as their esthetic value (Cupchik and Berlyne, 1979; Mullin et al., 2017; Verhavert et al., 2017; Schwabe et al., 2018). Examples of low-level features studied in affective pictures are image brightness (Lakens et al., 2013; Kurt et al., 2017), color (Bekhtereva and Muller, 2017) and spatial frequency content (Delplanque et al., 2007; De Cesarei and Codispoti, 2013; Muller and Gundlach, 2017). In a recent study, Rhodes et al. (2019) compared the Fourier amplitude spectra of aversive and neutral pictures. The authors showed that a support vector machine (SVM) can learn to discriminate between the two picture categories with an accuracy of 70%, based on the spectral amplitude information. However, because swapping amplitude spectra between picture categories did not affect the ratings, the authors concluded that the amplitude differences were actually not used by the human visual system to discriminate between the affective picture categories.

Less well investigated is the question of whether image properties of higher order, which reflect global image structure or image composition, may be involved in emotional processing of affective pictures. For example, a gun presented in a blurry, low-contrast and almost colorless photograph may evoke a higher aversive reaction than the same object presented in a attractive advertisement that is well balanced in brightness, color and image composition. By the same token, intrinsically pleasant scenes may be photographed in various ways that follow esthetic principles. As an example, erotic pictures, which are rated as positive and highly arousing, usually depict more or less symmetric bodies arranged in a stereotypically ordered manner. The influence of stimulus properties that are independent of specific content may be particularly relevant for studies demonstrating effects that arise as early as 100–200 ms during the neurophysiological processing of emotional stimuli (Junghöfer et al., 2001; Hindi Attar and Müller, 2012). To summarize and to put it simply, affective pictures are likely to evoke emotions not only by *what* they show, but also by *how* they show it.

The dichotomy between the processing of pictorial content and form has been an issue also in the field of experimental esthetics. On the one hand, it is clear that esthetic experience depends on image content, cultural context and the viewer’s familiarity and expertise, which are subject to cognitive processing to a large extent (Jacobsen, 2006; Cupchik et al., 2009; Redies, 2015). For example, Gerger et al. (2014) demonstrated that the esthetic judgments of both artworks and affective images depend on whether the stimuli are presented in an art context (“This is an artwork.”) as compared to a non-art context (“This is a press photograph.”). On the other hand, researchers have identified several formal image properties that are associated with visually pleasing images, such as high-quality photographs and artworks (Graham and Redies, 2010; Sidhu et al., 2018). Not surprisingly, many of these properties represent global features in images, i.e., they reflect the spatial arrangement of pictorial elements across the image (Brachmann and Redies, 2017). The search for stimulus properties that render stimuli visually pleasing originated in the 19th century, when the founder of experimental esthetics, Gustav Theodor Fechner, investigated whether human observers generally prefer rectangles whose sides follow the golden ratio (Fechner, 1876). However, his conclusions on the role of the golden ratio in visual preference was not confirmed by rigorous psychological testing (McManus et al., 2010). Recently, modern computational methods have allowed us to identify a number of global image properties that can be associated with visually pleasing images (for a review, see Brachmann and Redies, 2017). For example, some properties reflect summary statistics of luminance changes, such as edge complexity (Forsythe et al., 2011; Bies et al., 2016; Güclütürk et al., 2016), color statistics (Palmer and Schloss, 2010; Mallon et al., 2014; Nascimento et al., 2017), or particular Fourier spectral properties (Graham and Field, 2007; Redies et al., 2007b). Other properties describe the fractal nature and self-similar distribution of luminance and color gradients (Redies et al., 2012; Spehar et al., 2016; Sidhu et al., 2018; Taylor, 2002; Taylor et al., 2011) or other regularities in the spatial layout of such basic pictorial features across images (Brachmann et al., 2017; Redies et al., 2017). Several of the above-mentioned properties of pleasing images exhibit regularities that are shared by natural scenes (Graham and Field, 2007; Redies et al., 2007b, 2012; Nascimento et al., 2017; Taylor, 2002; Taylor et al., 2011), but differences between the image categories have also been described (Redies et al., 2007a; Schweinhart and Essock, 2013; Montagner et al., 2016). The human visual system is adapted to process the statistics of the natural environment efficiently (Olshausen and Field, 1996). Because natural and visually pleasing images share some formal characteristics, it has been speculated that efficient processing might be the basis of esthetic perception as well (Redies, 2007; Renoult et al., 2016). A causal link between some of the image properties and visual preference has been established experimentally (Schweinhart and Essock, 2013; Jacobs et al., 2016; Spehar et al., 2016; Nascimento et al., 2017; Grebenkina et al., 2018; Menzel et al., 2018). Interestingly, human observers often perceive images with Fourier spectra that deviate from natural (scale-invariant) statistics as unpleasant (Juricevic et al., 2010; O’Hare and Hibbard, 2011).

In summary, global image properties have been linked to visual preference and/or esthetic experience in diverse types of natural, artificial and artistic visual material. In the present study, we show that these properties can be used to predict emotional responses to affective pictures from five published datasets. The details of the datasets are listed in Table 1.

**TABLE 1:** Details of the affective image datasets analyzed.

Database	Image categories	*n*	Image size (pixel)	Rating terms	Rating scale	References
*International Affective Picture System* (IAPS)	Humans, animals, objects and scenes	1182	1024 × 768	valence, arousal dominance	1–9 (SAM)	Lang et al. (2008)
*Geneva Affective Picture Database*		728	640 × 480	valence, arousal	Continuous rating scale from 1 to100	Dan-Glauser and Scherer (2011)
	Subsets:					
(GAPED)	- animal mistreatments (GAPED-A)	124		external/internal norms		
	- human concerns (GAPED-H)	105		external/internal norms		
	- neutral (GAPED-N)	89				
	- positive (GAPED-P)	120				
	- snakes (GAPED-Sn)	132				
	- spiders (GAPED-Sp)	158				
*Nencki Affective Picture System* (NAPS)	People, faces, animals, objects and landscapes (NAPS-H)	1356	1600 × 1200	valence arousal avoiding/approaching behavior	1–9 steps	Marchewka et al. (2014)
	- set of fear-inducing pictures (NAPS-SFIP)	886	1024 × 768	valence arousal fear	1–5 (also with SAM)	Michalowski et al. (2017)
	- set of erotic pictures (NAPS-ERO)	200	1600 × 1200	- (no rating available)		Wierzba et al. (2015)
*Open Affective Standarized Image Set* (OASIS)	humans, animals, objects and scenes	900	500 × 400	valence arousal	1–7 (Likert scale)	Kurdi et al. (2017)
*Disgust-Related Images* (DIRTI)	Food, body products, (dead) animals, injuries/infections and hygiene	300	1024 × 768	valence arousal disgust fear	1–9 steps	Haberkamp et al. (2017)

*n, number of images; SAM, self-assessment manikin.*

One of the most widely used datasets in behavioral research is the *International Affective Picture System* (IAPS; Lang et al., 2008). It contains 1182 color pictures of pleasant, neutral and unpleasant content across the entire affective space, including human faces, landscapes, animals, various objects, erotica, press photographs of war and catastrophes, severe injuries, mutilation and corpses. The IAPS was established as a dataset freely available to researchers, in order to enable a comparison between studies. Together with each image, Lang et al. (2008) published ratings for valence (ranging from pleasant to unpleasant), arousal (ranging from calm to excited) and aspects of dominance or control. For an assessment of these three emotional dimensions, the authors used a *self-assessment manikin* (SAM). SAM is a non-verbal method that permits intercultural comparisons and the inclusion of participants at a very young age (Bradley and Lang, 1994). To complement and extend the IAPS database, e.g., to increase the number of images with specific content, a number of additional databases have been developed in recent years (e.g., for EEG studies; Dan-Glauser and Scherer, 2011; Horvat, 2017). In the present study, we did not attempt to analyze all of these datasets because they are too numerous and diverse. Instead, we focused on the following datasets (Table 1), which have been widely used in recent years.

The *Geneva Affective Picture Database* (GAPED; Dan-Glauser and Scherer, 2011) contains 730 pictures that focus on four specific negative contents: Spiders, snakes, and scenes that relate to violation of moral/ethical (internal) or legal (external) norms. Two other subsets depict images with neutral or positive content (Table 1). With the dataset, the authors provide scores for valence, arousal and for acceptability with respect to internal or external norms.

The *Nencki Affective Picture System* (NAPS; Marchewka et al., 2014) contains 1356 high-quality photographs divided into five categories (people, faces, animals, objects, and landscapes), which were rated according to their valence and arousal and along the approach-avoidance dimension. Moreover, some basic physical properties (luminance, contrast, complexity and entropy of the gray-level intensity histograms) are available for this dataset. The authors also published a partially overlapping dataset of 886 pictures, which focused on fear induction and can be used in phobia research (NAPS-SFIP; Michalowski et al., 2017), as well as a dataset of 200 unrated erotic pictures (NAPS-ERO; Wierzba et al., 2015).

The *Open Affective Standarized Image Set* (OASIS; Kurdi et al., 2017) comprises 900 color images in four distinct categories (humans, animals, objects and scenes) that cover a wide variety of themes and were rated for valence and arousal.

Finally, the *Disgust-Related Images* (DIRTI) constitute a dataset of 240 high-quality pictures that focus on disgust (Haberkamp et al., 2017) and were rated for valence, arousal, and fear. The authors proposed that this dataset might be particularly useful for psychiatric studies.

In the present study, we ask whether global image properties allow us to predict the ratings of the five affective picture databases introduced above. The set of predictors used in our study cover aspects of color, symmetry, complexity and self-similarity as well as the distribution and variances of color and luminance edges in the images. These properties were selected because they cover a wide range of global image features that were previously shown to be associated with esthetic judgments of images. In the present study, we investigate to what extent the five datasets differ in their image properties and whether particular patterns of image properties can predict the ratings of specific emotions. We use two independent approaches for this purpose. First, by using machine learning, we study to what degree a non-linear approach can predict valence and arousal ratings. Second, we use multiple linear regression to assess which linear combination of properties predicts the affective ratings best. We conclude by discussing the implications of our findings for future experimental studies using the affective picture datasets.

## Materials and Methods

### Affective Picture Datasets

The datasets were downloaded from the webpages mentioned in the original publications (DIRTI^1^, GAPED^2^, IAPS^3^, NAPS^4^, and OASIS^5^). The characteristics of the datasets are listed in Table 1. Two images from the GAPED database were not included in the analysis for technical reasons.

We considered it inappropriate to analyze the datasets together as one joint dataset because they differ in several important aspects. First, the scales for the rating terms (valence, arousal etc.) diverge between the datasets (Table 1). Second, we observed differences in the mean values for almost all image properties between the datasets (except for 2nd-order entropy; Table 2). Third, the correlations between the ratings vary between the datasets (Table 3).

**TABLE 2:** Mean values (±standard deviation) of the statistical image properties for each dataset of affective pictures.

Image property	IAPS (*n* = 1,182)	GAPED (*n* = 728)	NAPH-H (*n* = 1,356)	NAPS-SFIP (*n* = 886)	OASIS (*n* = 900)	DIRTI (*n* = 300)
H-channel***	0.300 (0.153)	0.296 (0.154)	0.316 (0.116)	0.317 (0.125)	0.291 (0.175)	0.270 (0.113)
S-channel***	0.451 (0.177)	0.327 (0.153)	0.524 (0.148)	0.349 (0.163)	0.338 (0.199)	0.282 (0.141)
V-channel***	0.510 (0.154)	0.524 (0.133)	0.332 (0.123)	0.529 (0.123)	0.521 (0.152)	0.600 (0.114)
Symmetry left/right***	0.449 (0.089)	0.480 (0.092)	0.465 (0.089)	0.481 (0.089)	0.467 (0.107)	0.474 (0.010)
Symmetry up/down***	0.418 (0.084)	0.451 (0.090)	0.419 (0.089)	0.427 (0.096)	0.418 (0.108)	0.453 (0.099)
Edge density***	101.07 (41.91)	100.38 (41.59)	108.62 (37.57)	110.81 (40.68)	99.79 (47.98)	93.14 (46.12)
Self-similarity***	0.609 (0.106)	0.692 (0.132)	0.629 (0.105)	0.634 (0.114)	0.609 (0.132)	0.637 (0.117)
Fourier slope***	−2.86(0.34)	−2.88(0.32)	−2.73(0.34)	−2.67(0.34)	−2.75(0.41)	−2.71(0.35)
Fourier sigma***	0.020 (0.027)	0.072 (0.055)	0.010 (0.011)	0.011 (0.012)	0.034 (0.029)	0.011 (0.013)
1st-order entropy**	4.28 (0.32)	4.25 (0.38)	4.25 (0.33)	4.23 (0.35)	4.23 (0.36)	4.27 (0.34)
2nd-order entropy	4.39 (0.19)	4.39 (0.22)	4.40 (0.17)	4.40 (0.17)	4.38 (0.19)	4.36 (0.23)
Variance Pa (× 10^–5^)***	25.03 (12.62)	22.99 (11.62)	22.06 (10.9)	22.95 (12.0)	29.21 (17.83)	23.06 (12.84)
Variance Pf (× 10^–5^)***	2.02 (0.66)	1.64 (0.67)	2.01 (0.67)	1.89 (0.68)	1.88 (0.76)	1.83 (0.72)

*Values differ significantly between datasets at ^∗∗^p < 0.01, ^∗∗∗^p < 0.001 (Kruskal-Wallis test, df = 5).*

**TABLE 3:** Mean, SD, Spearman Coefficients r (upper segments) and *p*-values (lower segments) for the subjective ratings of the affective image datasets.

Dataset	Range of Scale	Rating	Mean	SD	1	2	3	4
IAPS	1–9	1. Valence	5.03	1.77	-	**−0.24**	0.05	
		2. Arousal	4.81	1.15	<0.001	–	**−**0.02	
		3. Dominance	5.16	1.08	0.08	0.52	–	
GAPED	1–100	1. Valence	44.38	25.19	–	**−0.87**		
		2. Arousal	47.74	19.46	<0.001	–		
GAPED-A	1–100	1. Valence	21.26	12.40	–	**−0.80**	**0.83**	**0.74**
		2. Arousal	60.63	12.01	<0.001	–	**−0.70**	**−0.71**
		3. Int. Norms	26.98	13.94	<0.001	<0.001	–	**0.85**
		4. Ext. Norms	36.38	15.57	<0.001	<0.001	<0.001	–
GAPED-H	1–100	1. Valence	27.95	17.54		**−0.85**	**0.88**	**0.84**
		2. Arousal	58.71	15.02	<0.001	–	**−0.77**	**−0.81**
		3. Int. Norms	29.84	17.76	<0.001	<0.001	–	**0.92**
		4. Ext. Norms	35.42	17.44	<0.001	<0.001	<0.001	–
NAPS-H	1–9	1. Valence	5.39	1.62	–	**−0.79**	**0.97**	
		2. Arousal	5.10	1.06	<0.001		**−0.79**	
		3. AP-AV	5.36	1.48	<0.001	<0.001	–	
NAPS-SFIP	1–9	1. Valence	5.30	0.92	–	**−**0.03	**−0.50**	
		2. Arousal	1.46	0.50	0.30	–	**0.53**	
		3. Fear	1.05	0.13	<0.001	<0.001	–	
OASIS	1–7	1. Valence	4.33	1.23	–	0.002		
		2. Arousal	3.67	0.84	0.96	–		
DIRTI	1–9	1. Valence	4.43	1.61	–	**−0.92**	**−0.72**	**−0.97**
		2. Arousal	2.51	0.72	<0.001	–	**0.88**	**0.90**
		3. Fear	1.72	0.42	<0.001	<0.001	–	**0.73**
		4. Disgust	3.46	1.49	<0.001	<0.001	<0.001	–

*Bolded letters indicate significant correlations.*

### Image Properties

For each image, we calculated thirteen image properties that covered diverse aspects of global image structure, as described in the following paragraphs. These properties were selected from an even larger set of image properties, which our group has studied previously in visual artworks and other visually pleasing images (for references, see below). From the original set, we omitted properties that correlated strongly with each other (Braun et al., 2013; Redies et al., 2017; Brachmann, 2018). Overall correlations between the remaining properties used in the present study are listed in Supplementary Table S1 for all datasets together.

#### Color Values (HSV Color Channels)

Color plays an important role in human preference for images (Palmer and Schloss, 2010; Mallon et al., 2014; Nascimento et al., 2017), and in the rating of affective pictures (Bekhtereva and Muller, 2017). In the present study, color was analyzed in the Hue-Saturation-Value (HSV) color space, which consists of channels for hue (H), saturation (S), and value (V), respectively. In a previous study, our group demonstrated that the three channels in this space relate to the beauty ratings of abstract artworks (Mallon et al., 2014). We converted the original RGB-coded pictures into the HSV color space and calculated the average pixel value for each of the three channels by using the rbg2hsv algorithm of the MATLAB Toolbox (The MathWorks, Natick, MA, United States, Release 2012a).

#### Symmetry

Scientists and artists have claimed that symmetry is a fundamental and universal principle of esthetics, to which the human brain is particularly sensitive (for a review, see Bode et al., 2017). To measure symmetry, we used filter responses from the first layer of a convolutional neural network (CNN) that closely match responses of neurons in the visual cortex of higher mammals (Brachmann and Redies, 2016). This approach has the advantage that it captures a higher-order symmetry not only based on the color, edges and texture of images, but also on shapes and objects, thereby performing closer to human vision. We calculated left-right and up-down symmetry according to the algorithm provided by Brachmann and Redies (2016).

#### Edge Density

In general, human observers prefer an intermediate level of complexity in visual stimuli (Berlyne, 1971; Spehar et al., 2016), but there are large between-subject differences (Güclütürk et al., 2016). As a measure of image complexity, we summed up all edge responses in Gabor-filtered images in the present study, as described in detail by Redies et al. (2017). Our complexity measure correlates highly with other complexity measures that are based on luminance gradients and relate to subjective complexity (Braun et al., 2013).

#### Self-Similarity

Traditional Western oil paintings are characterized by an intermediate to high degree of self-similarity (Redies et al., 2012). In the present study, we calculated self-similarity with a derivative of the PHOG descriptor, which measures how similar the histograms of orientated gradients (HOGs; Dalal and Triggs, 2005) for parts of an image are compared to the histogram of the entire image. We reduced each image to 100,000 pixels size and used 16 equally sized orientation bins covering 360° for the histograms. Histograms at levels 1–3 were compared to the ground level histogram. For a detailed description of the method, see the appendix in Braun et al. (2013).

#### Fourier Slope

In radially averaged log-log plots of spectral power versus spatial frequency, the slope of a straight line (here called *Fourier slope*) is indicative of the relative strength of high spatial frequencies (fine detail) *versus* low spatial frequencies (coarse structure) of luminance changes across an image. In general, this slope is around -2 for natural scenes as well as for large subsets of artworks and other visually pleasing images. Human observers thus prefer statistics that are similar to those of natural scenes (Graham and Field, 2007; Redies et al., 2007b). We converted each image to grayscale with the Photoshop CS5 program and padded the images according to square ones, followed by Fast Fourier Transformation, as described in Redies et al. (2007b). After radially averaging the power spectrum, we plotted Fourier power versus spatial frequency. For equally spaced intervals in log-log space, the data points were averaged and fitted to a straight line by least-square fitting (Redies et al., 2007b).

#### Fourier Sigma

The deviation of the log-log Fourier power spectrum from a straight line is here called *Fourier sigma*. In most natural images and artworks, spectral power decreases linearly with increasing spatial frequency so that the Fourier sigma is small (Graham and Field, 2007; Redies et al., 2007b). Interestingly, larger values for Fourier sigma, i.e., larger deviations from a straight line, have been described for some unpleasant images (Fernandez and Wilkins, 2008; O’Hare and Hibbard, 2011). We calculated Fourier sigma as the sum of the squared deviations of the data points, which were binned in log-log space, from the fitted straight line, divided by the number of data points (Redies et al., 2007b).

#### First-Order and Second-Order Edge-Orientation Entropies

First-order entropy of edge orientations is a measure of how uniformly the orientations of luminance edges in an image are distributed across all orientations (Redies et al., 2017). If all orientations are represented at equal strength, first-order entropy is maximal. Values become smaller as particular orientations predominate the image. Second-order entropy is a measure of how independent or randomly edge orientations are distributed across an image. Values are close to maximal if edge orientations at given positions in an image do not allow any predictions of orientations at other positions of the image. Values for both entropies are high in some photographs of natural objects (for example, lichen growth patterns) and in artworks of different cultural provenance (Redies et al., 2017). Moreover, the edge-orientation entropies are predictors for esthetic ratings in diverse other types of man-made visual stimuli, for example, photographs of building facades or artificial geometrical and line patterns (Grebenkina et al., 2018). We calculated the two entropies by the method described in detail by Redies et al. (2017).

#### Variances of Feature Responses in Convolutional Neural Networks (CNNs)

The response characteristics of lower-layer CNN features resemble neuronal responses at low levels of the visual system, such as the primary visual cortex (Brachmann et al., 2017). The CNN features show regularities when responding to traditional artworks; they possess a high richness and variability, two statistical properties that can be expressed in terms of the variances P_a_ and P_f_, respectively (Brachmann et al., 2017). Richness implies that many features tend to respond at many positions in an image (low P_a_). Despite this overall richness, the feature responses are relatively variable between the sections of an image (high to intermediate P_f_) in traditional artworks. The two variances differ between artworks and several types of natural and man-made images. In the present study, we calculated the variances as described in Brachmann et al. (2017).

Code to calculate the above measures is available via the Open Science Framework^6^.

### Statistical Methods

#### Classification Analysis

To find out whether the set of image properties contains any information that contributes to the prediction of the affective ratings, we carried out a classification experiment using a SVM with a radial basis function (RBF) kernel. SVM is a widely-used machine-learning algorithm, which partitions the feature space of the input data by using hyperplanes in a way that maximizes the generalization ability of the classifier. We used the Scikit-learn library (Pedregosa et al., 2011) in Python to implement this classifier and compute the results.

The classifier was trained separately on each of the five datasets. The analysis was restricted to the two rating terms that were common to all five datasets (valence and arousal). For each dataset and rating term, the affective pictures were ranked according to the rating. Rated images were binned into three equally-sized clusters, which represented the pictures with the lowest ratings, intermediate ratings, and the highest ratings. The intermediate cluster was not used in the classification experiment. The SVM-RBF classifier was trained to distinguish between the pictures of low ratings and high ratings. A 10-fold cross-validation paradigm was used with 90% of the low/high rated images used for the training and 10% for testing in each round. Ten rounds of cross-validation with different partitions were performed. The validation results were averaged for each dataset and rating term separately and provided an estimate of the mean accuracy rate.

#### Multiple Linear Regression Analysis

We used multiple linear regression to determine the dependence of the ratings on the thirteen independent variables for each dataset. For this task, we used the lm package in the R project (R Development Core Team, 2017). *R*^2^ values were calculated for each image property to estimate how much of the variability in the outcome is mediated by the predictors of each model. *R*^2^ values were adjusted to account for the number of predictors in each model (*R*^2^_adj_). As an index for the effect of the independent variables on the outcome, we calculated standardized regression coefficients β_i_, which provide an estimate of the number of standard deviations, by which the outcome will change as a result of a change of one standard deviation in the predictor, assuming that the effects of all other predictors are held constant. Values for β_i_ were calculated with the lm.beta package of the R project.

Moreover, we aimed at reducing the number of independent variables in the multiple linear regression by excluding image properties that correlated highly. Using Akaike’s entropy-based Information Criterion (*AIC*), which considers the fit of the model as well as the number of parameters, we identified image properties that shared a similar prediction quality as other variables in the model. By a stepwise elimination, these variables were dropped from the model, as long as the model improved (i.e., the *AIC* value decreased). For the final models, *R*^2^_adj_ and b_i_ values were calculated again. The *R*^2^_adj_ values of the original and reduced (final) models were of similar magnitude, indicating that the predictive power was comparable.

#### Regression Subset Selection

Finally, as an alternative method to determine which of the variables plays the largest role in the different regression models, we carried out a regression subset selection with the *leaps* package of the R project (Miller, 2002). This algorithm performs an exhaustive search for the subset of variables that best predicts the model outcomes, without penalizing for model size. For each model size (between 1 and 13 predictors), we identified the variables in the 10 best models and plotted them in a single graph to visualize how often a given variable is predictor in the different models.

## Results

### Statistical Image Properties

The values for the global image properties of each image analyzed in the present study can be accessed at the Open Science Framework^7^. Mean values for the statistical image properties are listed in Table 2. Each of the image properties differs significantly between the five datasets, except for 2nd-order entropy. For example, differences between the databases are prominent between the NAPS-H and DIRTI datasets for the S-channel color values (*p* < 0.0001, Kruskal-Wallis test with pairwise Wilcox post-test, Bonferroni-Holmes corrected) and the V-channel values (*p* < 0.0001). This result implies that the DIRTI pictures are less saturated and lighter than the NAPS-H pictures on average. There is also a prominent difference between the NAPS-H and GAPED datasets in the Fourier sigma values (*p* < 0.0001). The GAPED pictures deviate more strongly from a scale-invariant Fourier spectrum than the NAPS-H pictures. Note that the image properties are not independent of each other and correlate to varying degrees, as revealed for the entire dataset of images in Supplementary Table S1. In general, color values (H-, S-, and V-channel), Fourier sigma and 1st-order entropy correlate weakly with the other variables (Spearman coefficients r smaller than 0.3). 1st-order entropy correlates strongly with 2nd-order entropy (r = 0.71). The two variances Pa and Pf correlate moderately and inversely with left/right symmetry (r = −0.50 and −0.65, respectively), up/down symmetry (r = −0.63 and −0.69), edge density (r = −0.59 and −0.46), and self-similarity (r = −0.70 and −0.61).

### Ratings

The ratings were taken from the five previous studies. They were obtained separately for each dataset using different scales (Table 1). Therefore, we cannot assume that the rating scales are comparable between datasets, even after normalization. As a consequence, we did not compare the ratings between the datasets and analyzed the relation between the dependent and independent variables within the datasets only.

Mean values for the affective ratings are listed in Table 3. Except for the NAPS-SFIP and OASIS datasets, the ratings for valence and arousal show negative correlations for the other datasets (r > −0.79; but r = −0.24 only for the IAPS dataset), confirming results from previous studies (Dan-Glauser and Scherer, 2011; Marchewka et al., 2014; Haberkamp et al., 2017). Some of the correlations between the other ratings are also of interest. For example, in the NAPS-H dataset, ratings for avoidance/approaching behavior correlate positively with valence (r > 0.97) and negatively with arousal (r > −0.79), as described by Marchewka et al. (2014). A similar pattern of dependency is found for ratings of acceptance of internal and external norms for the GAPED-A and GAPED-H subsets (Dan-Glauser and Scherer, 2011). An opposite pattern of dependency on arousal and valence ratings was observed for the fear ratings in the NAPS-SFIP dataset (Michalowski et al., 2017) and DIRTI dataset (Haberkamp et al., 2017), respectively, and for the disgust ratings in the DIRTI dataset (Table 3; Haberkamp et al., 2017).

### Classification

As a first step toward assessing whether the statistical image properties can predict emotional ratings of the affective pictures, we used a classification approach. An SVM-RBF classifier was trained to recognize images that belong to the one third of images with the highest ratings (*high*) and the one third with the lowest ratings (*low*), respectively. Ratings for valence and arousal were considered separately. In this binary task, the mean accuracy is 50% for a random assignment of the labels *high* and *low*. Results from a 10-fold cross-validation experiment (Table 4) reveal that mean classification accuracies for all datasets and each rating (valence and arousal) range from 57.4% ± 6.5 *SD* to 75.5% ± 12.3 *SD*. All mean accuracies are significantly higher than the random classification rate. We conclude that the image properties predict the ratings for valence and arousal in part. However, the prediction rates differ between the image datasets. For example, predictability is higher for the DIRTI dataset compared to the OASIS dataset for valence (75.5% vs. 58.7%; *t*[18] = 4.06, *p* = 0.0007) and for arousal (71.5% vs. 57.5%; *t*[18] = 3.27, *p* = 0.004).

**TABLE 4:** Mean accuracy of classifying pictures of low and high ratings for valence and arousal in each dataset (SVM-RBF classifier with 10-fold cross-validation).

Dataset	*Mean*	*95% CI*	*t*^1^	*p*^1^
**Valence**				
IAPS	58.2%	52.5–63.9%	3.44	0.0101
GAPED	65.1%	55.2–75.0%	3.62	0.0074
NAPS-H	64.3%	58.1–70.5%	5.23	0.0005
NAPS-SFIP	59.5%	51.2–67.8%	2.59	0.0291
OASIS	58.7%	55.3–62.0%	5.83	0.0003
DIRTI	75.5%	66.2–84.8%	6.20	0.0002
**Arousal**				
IAPS	59.3%	50.0–59.3%	3.82	0.0041
GAPED	62.4%	52.7–72.1%	2.90	0.0175
NAPS-H	57.4%	52.6–62.3%	3.45	0.0073
NAPS-SFIP	62.0%	59.0–65.1%	8.85	<0.0001
OASIS	57.5%	51.8–63.2%	2.97	0.0157
DIRTI	71.5%	63.1–79.9%	5.76	0.0003

*The by-chance classification accuracy is 50% for this binary task. ^1^ t statistics and p values for difference to random accuracy rate of 50% (two-sided one sample t-test, df = 9).*

### Regression Analysis

To investigate which of the image properties contributed to the prediction of the affective ratings, we subjected the data to a multiple linear regression analysis, considering the ratings as dependent variables and the image properties as independent variables.

To begin with, full models with all thirteen image properties were studied. Results are listed in Supplementary Tables S2, S3. For each model, we calculated *R*^2^ values, which indicate the percentage of predicted variance that is contributed by the image properties, and adjusted them to account for the number of predictors (*R*^2^_adj_). Values range from 0.017 to 0.195. Moreover, standardized regression coefficients β_i_ were calculated. In the tables, bold letters indicate the variables that have a significant effect on the ratings when the other variables are controlled for. Not all independent variables have the same predictive power in the full models. Therefore, to eliminate less influential variables from the models by stepwise iterations, we calculated the Akaike Information Criterion (*AIC*), which allows us to compare the relative quality of the fit for different original and reduced models, when applied to the same set of data (Akaike, 1974). Results for the reduced models are presented in Tables 5, 6. *R*^2^_adj_ values for the full and restricted models are of similar magnitude (range 0.020 to 0.195) for each dataset and rating. To simplify the description, we will explore the restricted models only in the following sections.

**TABLE 5:** Adjusted *R*^2^ Values and Standardized Regression Coefficients β_i_ for the IAPS, GAPED, NAPS-H and NAPS-SFIP datasets.

Variable/Parameter	IAPS (*n* = 1182)			GAPED *(all)* (*n* = 728)		NAPS-H (*n* = 1356)			NAPS-SFIP (*n* = 886)		
	**Valence**	**Arousal**	**Dominance**	**Valence**	**Arousal**	**Valence**	**Arousal**	**AV-AP**	**Valence**	**Arousal**	**Fear**
Adjusted *R*^2^	0.060***	0.087***	0.020*	0.190***	0.185***	0.141***	0.132***	0.146***	0.102***	0.076***	0.051***
*AIC*	1287.7	232.3	174.4	4556.5	4184.2	1125.3	**−**28.5	853.0	**−**235.6	**−**1296.1	**−**3664.8
H-channel				**0.117**	**−0.092**	**0.172**	**−0.160**	**0.174**	**0.139**	**−**0.049	**−0.079**
S-channel	**0.134**	0.044		**0.179**	**−0.084**	**0.253**	**−0.21**	**0.234**	**0.132**		**−**0.054
V-channel	**0.102**	0.046	**−**0.048	**0.132**	**−0.165**					0.071	
Symmetry left/right	0.060	**−0.116**	**−**0.065	0.095	**−**0.072	**0.164**	**−0.145**	**0.154**	**196**	**−0.105**	
Symmetry up/down	**−0.145**	**−0.097**		**−0.182**	0.079	**−0.146**	**0.148**	**−0.191**	**−0.145**	**−0.203**	
Edge density		**0.097**				**0.155**	**−0.078**	**0.159**	**0.127**	**−0.226**	**−0.193**
Self-similarity	0.076		**−0.065**	0.074			**−**0.072			0.111	
Fourier slope	**0.137**	**−0.153**		**0.101**	**−0.159**		**−**0.052		0.062		**−**0.055
Fourier sigma	**−0.098**	0.048		**−0.159**	**0.132**				**−0.092**		
1st-order entropy	**−0.095**	**0.105**	0.090		**0.088**		**0.137**	**−**0.04		**0.214**	**0.105**
2nd-order entropy		**0.127**	**−0.16**	0.062					**−**0.065	0.133	
Variance Pa	**0.112**		**0.14**	**0.171**	**−0.13**	**0.183**	**−0.154**	**0.191**	**0.142**	**−**0.088	**−0.119**
Variance Pf		**−0.141**		**0.128**	**−0.224**			**−0.062**		**−0.167**	

*Results are for models, in which the number of variables was reduced according to the Akaike Information Criterion (see section “Materials and Methods”). Bolded variables had a significant effect on the ratings when the other variables were controlled for. AIC, Akaike Information Criterion (compare to Supplementary Table S2). Significant at level ^∗^p < 0.05; ^∗∗^p < 0.01; ^∗∗∗^p < 0.001.*

**TABLE 6:** Adjusted *R*^2^ Values and Standardized Regression Coefficients β_i_ for the OASIS and DIRTI datasets.

Variable/Parameter	OASIS (*n* = 900)		DIRTI (*n* = 300)			
	**Valence**	**Arousal**	**Valence**	**Arousal**	**Fear**	**Disgust**
Adjusted *R*^2^	0.094***	0.154***	0.166***	0.195***	0.195***	0.194***
*AIC*	287.8	**−**459.7	237.9	**−**254.7	**−**576.9	182.5
H-channel	**−0.094**	**−**0.061				
S-channel	**0.163**	**0.119**	**0.231**	**−0.177**		**−0.184**
V-channel	**0.101**		**−0.171**	**0.214**	**0.289**	**0.208**
Symmetry left/right						
Symmetry up/down		**−0.027**	**−0.237**	**0.215**		**0.240**
Edge density				0.095		0.099
Self-similarity						
Fourier slope	**0.09**	**−0.225**				
Fourier sigma		**−0.092**		**−**0.085		**−**0.091
1st-order entropy	**0.183**	**0.173**	**−0.135**	**0.189**	**0.214**	**0.153**
2nd-order entropy	**−0.148**					
Variance Pa		**−0.084**				
Variance Pf	**0.21**	**−0.185**			**−0.238**	

*Results are for models, in which the number of variables was reduced according to the Akaike Information Criterion (see section “Materials and Methods”). Bolded variables had a significant effect on the ratings when the other variables were controlled for. AIC, Akaike Information Criterion (compare to Supplementary Table S3). Significant at level ^∗^p < 0.05; ^∗∗^p < 0.01; ^∗∗∗^p < 0.001.*

**FIGURE 1:**
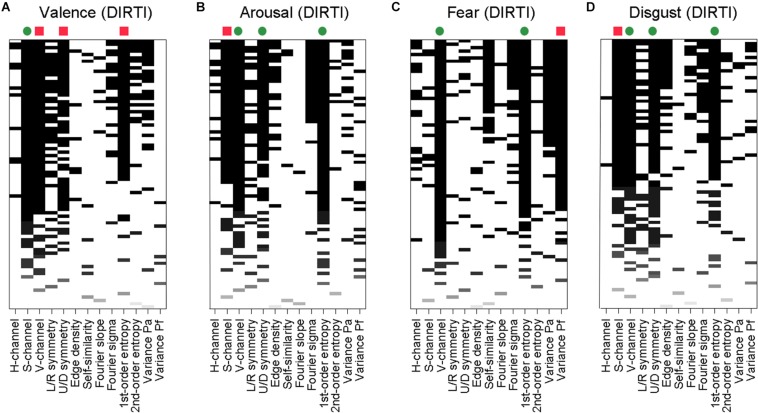
Results of regression subset selection for ratings of valence **(A)**, arousal **(B)**, fear **(C)**, and disgust **(D)** for the DIRTI dataset. Along each horizontal line in the graphs, results for one model are shown. Model size was varied systematically from 1 variable (bottom of the graphs) to all 13 variables (top). For each model size, the 10 models with the highest *R*^2^_adj_ values are represented. The image properties are indicated below the panels. The bars represent image properties that are predictors in the respective model. The intensity of the bar shadings indicate the magnitude of the *R*^2^_adj_ value of each model. On top of each graph, green dots and red squares indicate variables that were significant predictors with positive and negative effects on the ratings, respectively, in the multiple linear regression analysis (variables with bolded β_i_ values in Table 6).

Additionally, in view of the correlations between some of the independent variables (Supplementary Table S1), we asked how much arbitrariness is reflected in the specific subsets of image properties that were selected for the reduced models in the multiple linear regression analysis. We therefore used regression subset selection as another method to identify the most predictive subset of image properties by an exhaustive search (Miller, 2002). Exemplary graphical representations of the results for the DIRTI datasets are shown in Figure 1. Results for the other datasets are visualized in Supplementary Figures S1–S3. In the plots, more solid black columns indicate variables that play a role in a larger number of models. A comparison with the results from multiple linear regression (Tables 5, 6) reveals that both types of analyses converge on a similar set of predictors. This convergence indicates that the variables selected in the multiple linear regression analysis represent the most predictive ones indeed.

We observe a high variability in the image properties that predict the ratings in the different datasets in the following respects: First, the datasets differ in the image properties that predict their ratings. Second, image properties differ in which of the individual ratings they predict within a given dataset. All variables predict ratings for some of the datasets, but some variables, such as the color parameters (H-, S-, and V-channel), 1st-order entropy, the symmetry measures (left/right symmetry and up/down symmetry), and the CNN variances (Pa and Pf) serve as predictors in all datasets, albeit for different ratings. None of the image properties is a significant predictor for all ratings over all five datasets.

**FIGURE 2:**
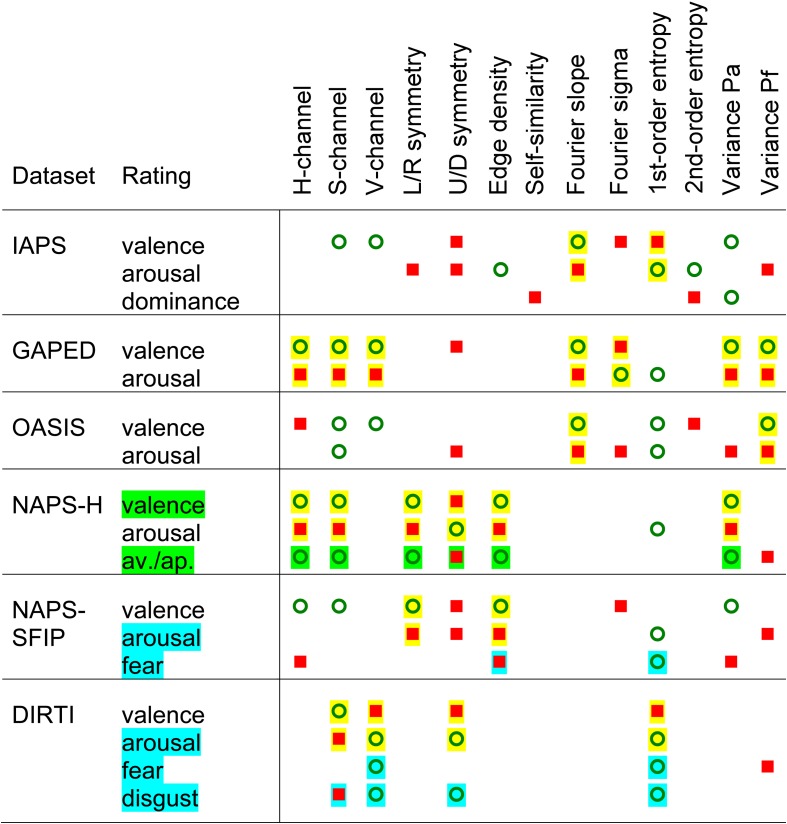
Schematic diagram of the results from the linear regression analysis with all 13 image properties (as indicated on top) for the ratings of different datasets (as indicated on the left-hand side). Results for the reduced models are shown, and only for properties that had a significant effect on the ratings when the other variables were controlled for (bolded variables in Tables 5, 6). The symbols indicate image properties that correlate positively (green circles) or negatively (red squares) with the respective rating. The yellow shadowing indicates image properties with opposite effects on the ratings of valence and arousal. The green shadowing for the NAPS-H dataset marks image properties with similar predictive effects on the ratings of valence and avoidance/approach, respectively. The cyan shadowing for the NAPS-SFIP and DIRTI dataset marks image properties with similar predictive effects on the ratings of arousal, fear and disgust, respectively.

Interestingly, the independent variables contribute to the ratings of valence and arousal with opposite algebraic signs for three of the datasets (see b_i_ values in Tables 5, 6). The positive and negative effects on the ratings are indicated by green dots and red squares, respectively, on top of the predictors in Figure 1 and Supplementary Figures S1–S3. A graphical synopsis of these results is provided in Figure 2. An opposite predictive pattern for the valence and arousal ratings is observed for the DIRTI dataset (Figure 1 and Table 6), the GAPED dataset (Supplementary Figures S2A,B and Table 5), and the NAPS-H dataset (Supplementary Figure S3 and Table 5). The ratings for valence and arousal correlate strongly and inversely for each of these datasets (Table 3), as reported previously (Marchewka et al., 2014; Haberkamp et al., 2017). For the NAPS-H dataset, the ratings along the dimension of avoidance/approach are predicted by a pattern of variables similar to the valence ratings (Supplementary Figures S3A,C). Also, the ratings correlate strongly with each other (Table 3; Marchewka et al., 2014). The fear and disgust ratings for the DIRTI dataset show a pattern of predictors similar to those of the arousal ratings (Figures 1B–D, 2; for correlation coefficients, see Table 3; Haberkamp et al., 2017).

The GAPED database (Dan-Glauser and Scherer, 2011) contains 6 subsets of different content (89–158 pictures per subset). We analyzed all subsets together (Table 5), but also each subset separately (Supplementary Table S4). Probably because of the limited number of pictures per category, the predictive power of the image properties reached significance for a few of the image properties only in the subsets. Nevertheless, the subset analysis sheds some additional light on the differences between the affective image categories. The image properties have relatively low predictive power for ratings of valence and arousal (*R*^2^_adj_ values between 0.022 and 0.076) for the GAPED-A (animals mistreatment scenes) subset, the GAPED-Sn (snakes) subset and the GAPED-Sp (spiders) subset. Predictive power is higher for the GAPED-H (scenes violating human rights) subset (*R*^2^_adj_ values of 0.223 for valence and 0.194 for arousal). For the GAPED-A and GAPED-H datasets, ratings of acceptability with respect to internal (moral) and external (legal) norms (Dan-Glauser and Scherer, 2011) were analyzed in addition. The predictors for these ratings are largely shared with the valence and arousal ratings, with the same algebraic sign for the valence ratings and an opposite sign for the arousal ratings (Supplementary Table S4).

## Discussion

We studied to what extent global image properties predict emotional responses to stimuli from five affective picture datasets (IAPS, GAPED, NAPS, OASIS, and DIRTI). The datasets were analyzed separately because they differ in important respects (see section “Materials and Methods”). Nevertheless, the datasets share some features, as outlined below.

The present study confirms previous findings that some of the ratings are correlated with each other in a given database (Dan-Glauser and Scherer, 2011; Haberkamp et al., 2017; Kurdi et al., 2017; Lang et al., 2008; Marchewka et al., 2014). In particular, the valence ratings correlate inversely with the arousal ratings in the IAPS, GAPED, NAPS-H, and DIRTI datasets in general (Figure 2 and Table 3). Moreover, the valence or arousal ratings correlate also with some of the other the ratings (Figure 2 and Table 3). In particular, ratings of internal/external norms (GAPED-A and GAPED-H; Dan-Glauser and Scherer, 2011) and approach/avoidance (NAPS-H; Marchewka et al., 2014) correlate positively with valence ratings. By contrast, ratings of fear or disgust (NAPS-SFIP, DIRTI; Marchewka et al., 2014; Haberkamp et al., 2017) correlate positively with arousal ratings. These correlations are mirrored by similar sets of predictive image properties (Figure 2 and Tables 5, 6). As expected, if the correlation between two ratings is negative for a given dataset, the predictive properties tend to have regression coefficients β_i_ of opposite algebraic signs. With a positive correlation, the regression coefficients β_i_ tend to have the same algebraic sign. For the IAPS and OASIS datasets, such systematic relations are not observed, as correlations between the ratings are weaker or absent (Table 3).

### Prediction of Affective Ratings by Global Image Properties

The image properties studied by us have been associated with preference judgments in previous studies (see section “Introduction”). We therefore speculated that they might predict emotional responses, such as valence and arousal, as well. The results from the present study confirm this notion in general.

We made use of two different methods to examine whether the image properties can predict the ratings. To start with, we used deep learning with an SVM-RBF classifier in a binary task, in which pictures with high versus low ratings for valence and arousal had to be distinguished (Table 4). The obtained classification rates differ between the datasets. For example, predictive power is relatively low for the IAPS dataset (58.2 and 59.3% classification rate for valence and arousal, respectively), but high for the DIRTI dataset (75.5 and 71.5%). Moreover, to specify which of the image properties has an effect on the ratings, we carried out linear regression analyses. The percentage of predicted variability (*R*^2^_adj_) ranges from 2 to 20%. Again, the predictive power differs between datasets. For example, it is relatively low for the ratings of the IAPS dataset (2.0–8.7%; Table 5), compared to the DIRTI dataset (16.6–19.5%; Table 6). These results imply that global perceptual cues in the IAPS pictures predict the affective ratings less strongly than the pictures of the DIRTI dataset. In other words, the images of the IAPS dataset are more balanced with regard to their image properties and, consequently, formal image structure represents less of a potential confounding factor in the evaluation of the emotional content than for the DIRTI database.

The origin of the correlations between image properties and affective ratings is unclear. One possibility is that people, who photograph or select pictures for affective datasets, (un)consciously choose pictures that are congruent at the perceptual and semantic levels. For example, someone might take a photograph of a pleasing landscape by carefully selecting a well-balanced and appealing detail of the scene while a photograph of vomit in a dirty sink might be less esthetically motivated and composed. In a similar vein, Sammartino and Palmer (2012) postulated that people prefer images if their spatial composition optimally conveys an intended or inferred meaning of the image (“representational fit”), which enhances their esthetic impact.

The magnitude of the present results can be compared to ratings in the field of visual esthetics. Here, sets of objective image properties similar to the ones used in the present study have been used to predict diverse esthetic ratings, such as linking, beauty or visual preference. As in the present study, results depend on the datasets analyzed. For example, in the study by Sidhu et al. (2018), predicted variances ranged from 4% (for beauty ratings of abstract art) to 30% (for liking rating of representational art). Grebenkina et al. (2018) reported predicted variances between 5% (for pleasing ratings of CD album covers) and 55% (for liking ratings of building facade photographs). Schwabe et al. (2018) analyzed abstract artworks and non-artistic images and obtained predicted variances that ranged from 27 to 46% for ratings of how harmonious and ordered the images were, respectively. The variances predicted in the present study are thus comparatively low to moderate (up to 20%), depending on the dataset analyzed.

Evidence for a direct role of low-level image properties in affective evaluations of images has been obtained, for example, by Bekhtereva and Muller (2017) who found that picture color can facilitate higher-level extraction of emotional content. In this context, it is of interest that the cortical representation of emotional categories, such as fear, anger and desire, was recently shown to be intertwined with the processing of visual features in visual cortical areas (V1–V4) (for example, see Kragel et al., 2019). One possible interpretation of this finding is that low-level visual features are directly associated with the processing of distinct emotion categories already at the level of visual cortex (Sabatinelli et al., 2004; Kragel et al., 2019). Our finding that specific combinations of image properties can be linked to different affective ratings is compatible with this notion.

Studies regarding scene perception reached similar conclusions. Short and masked presentations of 100 ms and less are sufficient for viewers to comprehend and describe complex scenes such as line drawings, but also naturalistic scenes (Dobel et al., 2007; Glanemann et al., 2016; Zwitserlood et al., 2018). This finding applies even to scenes containing interacting persons. Although the gist (a coarse understanding and categorization of the scene as a whole) of a scene and its coherence can be rapidly extracted using features such as body orientation of involved agents, a more refined semantic analysis of a scene requires additional processing. Most authors in this field agree that a first sweep of feed-forward processing can account for the high ability to categorize and sometimes even recognize complex images (Potter et al., 2014; Wu et al., 2015), but that reentrant processing from higher cognitive functions is necessary for representations in high detail. As a neural mechanism underlying interactions between objective image properties and subjective (cognitive) evaluations, the “multiple waves” model proposed by Pessoa and Adolphs (2006) is compatible with our assumptions. In this model, multiple pathways of processing become activated simultaneously already at an early stage of visual processing. By extensive feedback loops, the pathways enable a “complex ebb-and-flow of activation” thereby “sculpting” (p. 19) the activation profile of a specific stimulus throughout the visual cortex and the amygdala. In this vein, the proposed image properties evoke an initial activation profile, which, at later stages, becomes refined by higher-order cognitive processes. It was proposed for overlearned emotional stimuli, such as emotional words, that the outer appearance of a word is tightly linked to an “emotional tag” triggering processes of emotional attention (Roesmann et al., 2019). Similarly, the here-described image properties could trigger initial processing channels that operate in parallel, which in turn activates higher cognitive functions.

Similarly, low-level image properties were shown to play a role in esthetic judgments. A particularly well-studied example is the preference of curved over angular objects or line patterns (Bar and Neta, 2006; Palumbo et al., 2015). This preference can be observed in different cultures (Gómez-Puerto et al., 2017) and was even demonstrated in great apes (Munar et al., 2015). Another example is the observation that the spatial frequency content of face images and their surround has an effect on ratings of face attractiveness (Menzel et al., 2015). In the color domain, Nascimento et al. (2017) studied visual preferences for paintings with different color gamuts and found maximal preference for images with color combinations that matched the artists’ preferences, suggesting that artists know what chromatic compositions observers like.

Besides general effects on esthetic preference, there are also indications that individual taste for low-level features plays a role in esthetic judgments. Mallon et al. (2014) examined beauty ratings in abstract artworks, using a set of image properties that also included several of the present variables. The authors showed that color values in particular are relatively good predictors of the beauty ratings in general. Correlations became stronger after participants had been clustered in groups with similar preferences, suggesting that individual “taste” for specific image properties contributes to esthetic judgments. Preferences for patterns with different degrees of complexity are also subject to individual variability. While most participants like images of intermediate complexity, subgroups of participants prefer images of high and low complexity, respectively (Güclütürk et al., 2016; Spehar et al., 2016; Viengkham and Spehar, 2018). In studies of affective pictures, clustering has been applied to the selection of representative stimuli from the IAPS dataset (for example, see Constantinescu et al., 2017) but, to our knowledge, not to groups of observers, perhaps because emotional reactions are considered less prone to individual variability than esthetic judgments.

### Datasets Differ in Which Image Properties Predict the Affective Ratings

We also noted that the datasets differ widely in how many and which of the image properties are predictive for the ratings, when the other image properties are accounted for (bold regression coefficients in Tables 5, 6). This variability can be readily appreciated in the plots of the regression subset selection analysis (Figure 1 and Supplementary Figures S1–S3), as summarized in Figure 2. For example for the IAPS dataset, almost all image properties contribute to one or more of the ratings, except for the H-channel. Thus, the relatively low percentage of predicted variability of this dataset (*R*^2^_adj_ values of 0.02–0.09) is associated with many different variables. By contrast, only 4 out of the 13 properties are predictive for the DIRTI dataset (2 color values, up/down symmetry and 1st-order entropy). The relatively large *R*^2^_adj_ values for the DIRTI dataset (0.17–0.20) are thus mediated by a few image properties only.

The image properties also differ in how many datasets they are associated with. For example, self-similarity weakly predicts the dominance rating in the IAPS dataset only. Other image properties, such as the S-channel value, up/down symmetry and 1st-order entropy of edge orientations, are associated with specific ratings in all subsets.

Makin (2017) alluded to the multiplicity and variability of image features that determine esthetic preferences as the “gestalt nightmare” because the different properties are not orthogonal to each other and differentially interact to mediate esthetic perception, depending on the types of stimuli studied. The present results are compatible with this notion. Despite the large overall variability, we observe the following regularities.

First, a larger S channel value, i.e., more saturated colors, correlates with positive ratings for valence in all datasets. This finding is reminiscent of findings in experimental esthetics where diverse aspects of color perception play a prominent role in preference judgments (Palmer and Schloss, 2010; Mallon et al., 2014; Nascimento et al., 2017), in particular, if emotional terms are used in the esthetic ratings (Lyssenko et al., 2016). Second, a larger 1st-order entropy of edge orientations coincides with higher arousal ratings in all datasets. This measure assumes high values in traditional artworks (Redies et al., 2017) and is positively correlated with ratings for pleasing and interesting in photographs of building facades, but less so in other visual patterns, such as music CD covers (Grebenkina et al., 2018). Third, the left/right and up/down symmetry ratings correlate with valence and arousal ratings in all datasets, underlining the importance of (a)symmetry in esthetic perception (Jacobsen and Höfel, 2002; Gartus and Leder, 2013; Wright et al., 2017). Specifically, a more balanced up/down symmetry correlates with lower valence ratings (except for the OASIS dataset). The other dependencies are more erratic with no clear pattern of correlations across the datasets.

In conclusion, the affective picture datasets differ widely in their low-level perceptual qualities, partially precluding a direct comparison of the results across the different datasets, with the exception of the S-channel value, 1st-order entropy and up/down symmetry. This variability might be caused by biases in the selection of the pictures, different photographic techniques as well as differences in image content.

It should be stressed the present study is descriptive and does not address the question of whether any of the image properties actually induce specific emotions or are used to recognize affective content of pictures. Indeed, the association of an image property with a specific rating can be coincidental, as Rhodes et al. (2019) recently demonstrated for the slope of the Fourier spectrum (see section “Introduction”). An open question is to what degree image properties can predict complex evaluative processes in principle. It is very likely that there are many other predictive image properties that have not yet been described. Can future researchers predict viewer’ ratings with a much higher confidence by taking into account even more image properties? We doubt that this is the case because we assume, in line with most other researchers, that individual subjective factors like familiarity with stimuli, cultural influences, emotional states as well as personality trait will also mediate how an individual evaluates a specific image, in addition to objective image properties.

### Recommendations for Experimenters

As outlined above, the described image properties have an impact on valence, arousal and other affective ratings. We therefore consider it necessary to control for these factors in research that addresses emotional stimulus processing. Of the databases analyzed in the present study, the IAPS database had a relatively small impact of stimulus properties on the affective ratings and thus recommends itself.

An alternative approach would be to use the established values of individual pictures as covariates for statistical analyses. To foster such an approach, we are making the values available to the scientific community via the Open Science Framework (accessible at https://osf.io/r7wpz). Similarly, the provided values could be used across databases to generate picture sets (e.g., of positive versus negative valence) that are matched for the image properties with a prominent effect on the ratings. This way, a bias for particular image properties can be avoided when subsets of images are selected from a database.

Moreover, when images for novel databases are collected, we suggest that researchers establish the described image properties to control for them and/or to keep their impact on ratings low. The necessary methods and codes are all open source (see section “Materials and Methods”). Last but not least, the effect of image properties on affective ratings might be more prominent if the idiosyncratic style of one or a few photographers predominates in a given affective dataset. Presumably, such stylistic particularities can be avoided by collecting images from a wide range of sources and photographers.

In conclusion, the interplay between low-level image properties and their interaction with higher cognitive functions is a key issue in understanding emotional and esthetic perception. Here, we stress the impact of global image properties on emotional ratings and that this should be regarded in future research by selecting appropriate images from datasets. Additionally, we show how insights from empirical esthetics may shed light on basic visual and emotional perception.

## Data Availability Statement

The values for the global image properties of all five afffective image datasets can be accessed at the Open Science Framework (https://osf.io/r7wpz). For availability of code to calculate the properties, see text footnote 6.

## Author Contributions

CR, AK, and CD conceived the experiments. CR, MG, MM, and AK collected and analyzed the data. CR and CD wrote the manuscript.

## Conflict of Interest

The authors declare that the research was conducted in the absence of any commercial or financial relationships that could be construed as a potential conflict of interest.

## Footnotes

1 https://zenodo.org/record/167037#.XegL5y2×9E4

2 https://www.affective-sciences.org/researchmaterial

3 https://csea.phhp.ufl.edu/Media.html

4 http://naps.nencki.gov.pl

5 https://db.tt/yYTZYCga

6 https://osf.io/p6nuq, ./csvta, ./bd8ma, and ./xb983

7 https://osf.io/r7wpz
