# Supplementary material for: Global Image Properties Predict Ratings of Affective Pictures
Source: Front Psychol. 2020 May 12;11:953. doi: 10.3389/fpsyg.2020.00953 (PMC7235378; doi:10.3389/fpsyg.2020.00953)
Supplement: Supplementary file 1 [file Data_Sheet_1.PDF]

Supplementary Material

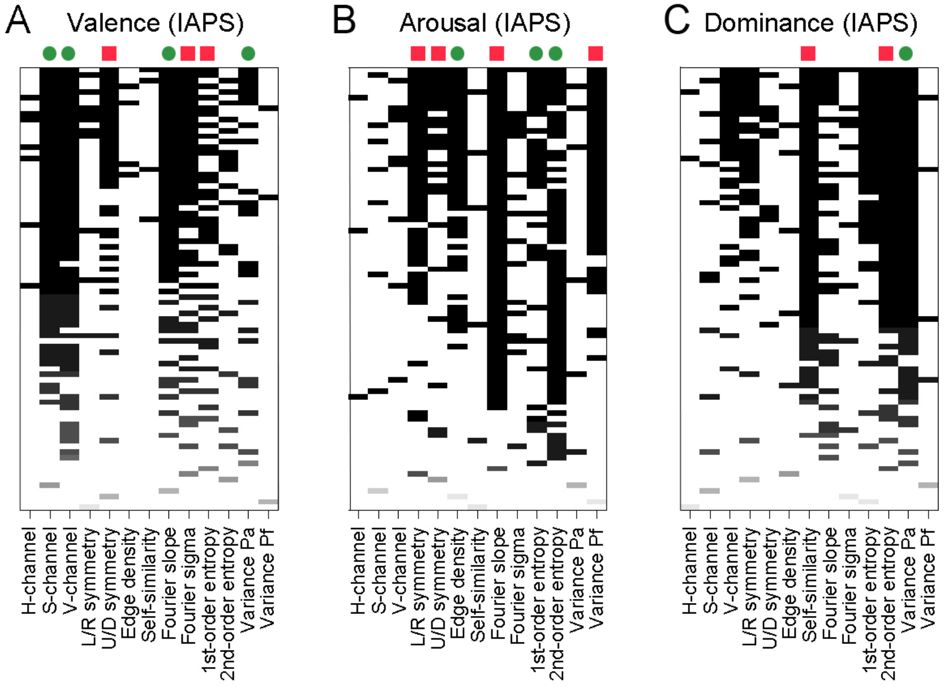

Supplementary Figure 1. Results of regression subset selection for ratings of valence (A), arousal (B) and dominance (C) for the IAPS dataset. Along each horizontal line in the graphs, results for one model are shown. Model size was varied systematically from 1 variable (bottom of the graphs) to all 13 variables (top). For each model size, the 10 models with the highest R^2_{adj} values are represented. The image properties are indicated below the panels. The bars represent image properties that are predictors in the respective model. The intensity of the bar shadings indicate the magnitude of the R^2_{adj} value of each model. On top of each graph, green dots and red squares indicate variables that were significant predictors with positive and negative effects on the ratings, respectively, in the multiple linear regression analysis (variables with bolded β_i values in Table 5).

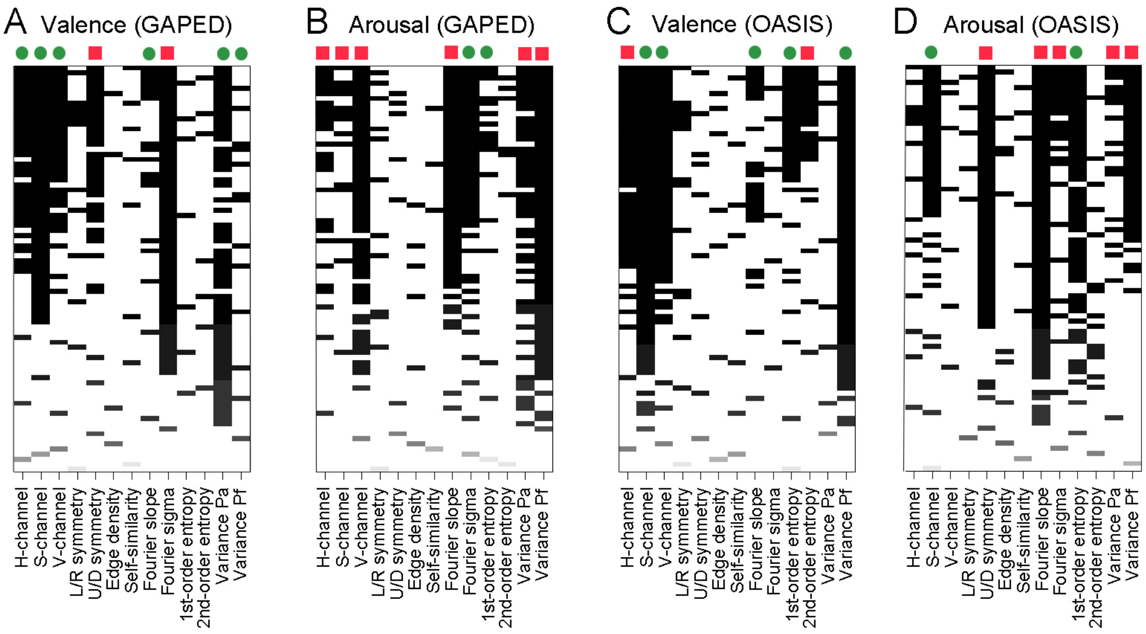

Supplementary Figure 2. Results of regression subset selection for ratings of valence (A, C) and arousal (B, D) for the GAPED dataset (A, B) and the OASIS dataset (C, D). Along each horizontal line in the graphs, results for one model are shown. Model size was varied systematically from 1 variable (bottom of the graphs) to all 13 variables (top). For each model size, the 10 models with the highest R^2_{adj} values are represented. The image properties are indicated below the panels. The bars represent image properties that are predictors in the respective model. The intensity of the bar shadings indicate the magnitude of the R^2_{adj} value of each model. On top of each graph, green dots and red squares indicate variables that were significant predictors with positive and negative effects on the ratings, respectively, in the multiple linear regression analysis (variables with bolded β_i values in Tables 5 and 6).

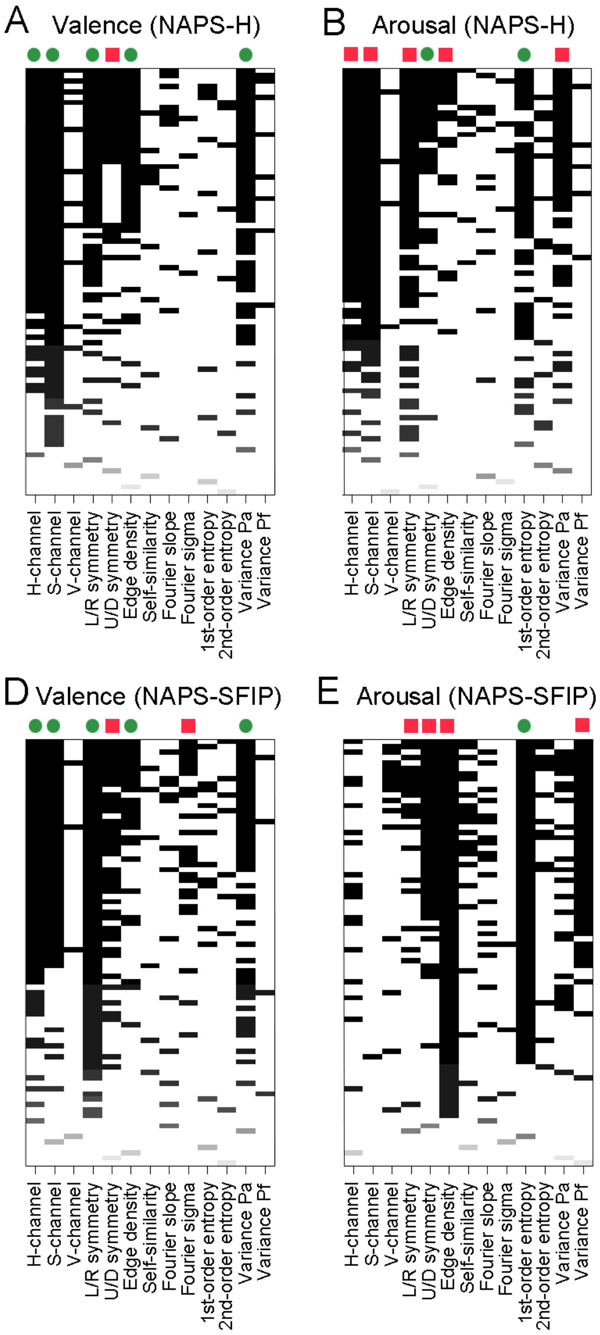

Supplementary Figure 3. Results of regression subset selection for ratings of valence (A, D), arousal (B, E), avoidance/approaching behavior (C) and fear (F) for the NAPS-H dataset (A-C) and the NAPS-SFIP dataset (D-F). Along each horizontal line in the graphs, results for one model are shown. Model size was varied systematically from 1 variable (bottom of the graphs) to all 13 variables (top). For each model size, the 10 models with the highest R^2_{adj} values are represented. The image properties are indicated below the panels. The bars represent image properties that are predictors in the respective model. The intensity of the bar shadings indicate the magnitude of the R^2_{adj} value of each model. On top of each graph, green dots and red squares indicate variables that were significant predictors with positive and negative effects on the ratings, respectively, in the multiple linear regression analysis (variables with bolded β_i values in Tables 5 and 6).

Supplementary Table 1

Spearman Coefficients ρ for Correlations Among Statistical Image Properties. Results are for all images in the datasets listed in Table 1 (n=4468), except for the NAPS-SFIP and NAPS-ERO datasets.

	1	2	3	4	5	6	7	8	9	10	11	12	13
1. H-channel	-												
2. S-channel	-.19***	-											
3. V-channel	-.02	-.18***	-										
4. Symmetry (left/right)	.03*	.03	.04**	-									
5. Symmetry (up/down)	-.034**	.05***	-.03*	.58***	-								
6. Edge density	-.01	0.03*	-.10***	.42***	.49***	-							
7. Self-similarity	-.04**	.05***	-.08***	.53***	.71***	.53***	-						
8. Fourier slope	.11***	-.07***	.01	.52***	.39***	.46***	.33***	-					
9. Fourier sigma	-.04**	-.01	-.05**	.14***	.12***	.06***	.07***	-.01	-				
10. First-order entropy	-.05***	.14***	-.05***	.06***	.20***	.11***	.23***	.04**	-.08***	-			
11. Second-order entropy	.02	.10***	-.10***	.29***	.35***	.44***	.44***	.34***	-.02	.71***	-		
12. Variance Pa	.04**	-.02	.29***	-.50***	-.63***	-.59***	-.70***	-.53***	.02	-.20***	-.47***	-	
13. Variance Pf	-.03*	.10***	.05***	-.65***	-.69***	-.46***	-.61***	-.45***	-.19***	-.19***	-.43***	.62***	-

Note. Correlation significant at level * $p < .05$, ** $p < .01$ and *** $p < .001$

Supplementary Table 2

Adjusted R^2 Values and Standardized Regression Coefficients β_i for the IAPS, GAPED, NAPS-H and NAPS-SFIP datasets. Results are for full models with all thirteen independent variables. Bolded variables had a significant effect on the ratings when the other variables in the respective model were controlled for.

Parameter/ Variable	IAPS *(all)* (*n* = 1182)			GAPED *(all)* (*n* = 728)		NAPS-H (*n* = 1356)			NAPS-SFIP (*n* = 886)		
Rating	Valence	Arousal	Dominance	Valence	Arousal	Valence	Arousal	AV-AP	Valence	Arousal	Fear
Adjusted R^2	.069***	.087***	.017**	.187*	.184***	.139***	.130***	.144***	.098***	.074***	.047***
AIC	1291.4	239.7	185.7	4560.5	4187.4	1134.2	-22.0	861.6	-228.0	-1291.6	-3654.0
H-channel	.036	.020	-.034	**.118**	**-.092**	**.172**	**-.162**	**.173**	**.141**	-.049	**-.083**
S-channel	**.137**	.054	.171	**.179**	**-.081**	**.264**	**-.204**	**.236**	**.133**	-.011	-.053
V-channel	**.101**	.072	-.037	**.132**	**-.163**	.032	.016	.013	.012	.07	.006
Symmetry left/right	.071	**-.118**	-.05	.094	-.083	**.161**	**-.159**	**.185**	**.198**	**-.082**	-.016
Symmetry up/down	**-.141**	**-.09**	-.03	**-.183**	.062	**-.16**	**.145**	**-.181**	**-.147**	**-.192**	-.041
Edge density	.044	**.087**	-.011	.005	.041	**.158**	**-.081**	**.154**	**.128**	**-.219**	**-.202**
Self-similarity	.083	-.037	**.171**	.073	.012	-.007	-.079	-.011	.017	.100	.045
Fourier slope	**.129**	**-.166**	-.032	**.102**	**-.153**	-049	-.06	.014	.066	-.040	-.057
Fourier sigma	**-.109**	.050	.278	**-.159**	**.13**	.001	-.019	-.015	**-.091**	-.025	.017
1st-order entropy	**-.061**	**.110**	.084	.012	**.178**	-.042	**.106**	-.071	-.002	**.201**	**.173**
2nd-order entropy	-.046	**.117**	**-.149**	.519	-.104	.025	.03	.034	-.062	-.124	-.088
Variance Pa	**.113**	-.071	**.126**	**.171**	**-.132**	**.163**	**-.159**	**.185**	**.14**	-.088	**-.108**
Variance Pf	.047	**-.140**	-.009	.129	**-.221**	-.043	-.021	-.054	.023	**-.17**	-.06

Notes. *AIC*, Akaike Information Criterion (compare to Table 5). Significant at level *, $p < .05$; **, $p < .01$; ***, $p < .001$

Supplementary Table 3

Adjusted R^2 Values and Standardized Regression Coefficients β_i for the OASIS and DIRTI datasets. Results are for full models with all thirteen independent variables. Bolded variables had a significant effect on the ratings when the other variables in the respective model were controlled for.

Parameter/ Variable	OASIS (n = 900)		DIRTI (n = 300)			
Rating	Valence	Arousal	Valence	Arousal	Fear	Disgust
Adjusted R^2	.091***	.151***	.169***	.191***	.189***	.195***
AIC	296.9	-451.5	245.2	-246.5	-565.2	189.0
H-channel	**.095**	-.057	-.046	.051	.058	.017
S-channel	**.159**	**.124**	**.222**	**-.159**	-.043	**-.209**
V-channel	**.099**	-.005	**-.257**	**.267**	**.344**	**.252**
Symmetry left/right	.051	-.040	-.138	.095	.038	.137
Symmetry up/down	-.072	**-.247**	**-.188**	**.176**	.092	**.283**
Edge density	-.004	-.041	-.072	.100	.049	.149
Self-similarity	.049	.003	.122	-.123	-.196	-.120
Fourier slope	**.083**	**-.205**	.065	-.061	-.016	-.102
Fourier sigma	.013	**.008**	.082	-.095	-.085	-.101
1st-order entropy	**.207**	**.167**	**-.201**	**.242**	**.219**	**.22**
2nd-order entropy	**-.167**	-.00009	.115	-.083	-.023	-.073
Variance Pa	-.003	**-.089**	.177	-.133	-.170	-.122
Variance Pf	**.219**	**-.202**	-.04	-.042	**-.157**	.113

Notes. *AIC*, Akaike Information Criterion (compare to Table 6). Significant at level *, $p < .05$; **, $p < .01$; ***, $p < .001$

Supplementary Table 4

Adjusted R^2 Values and Standardized Regression Coefficients β_i for the GAPED subsets. Results are for models, in which the number of variables was reduced according to the Akaike Information Criterion (see Methods). Bolded variables had a significant effect on the ratings when the other variables were controlled for.

Variable	GAPED-A (n = 124)				GAPED-H (n = 105)				GAPED-N (n =89)		GAPED-P (n = 120)		GAPED-Sn (n = 132)		GAPED-Sp (n = 158)	
	Val.	Arousal	Int.	Ext.	Val.	Arousal	Int.	Ext.	Val.	Arousal	Val.	Arousal	Val.	Arousal	Val.	Arousal
Adjusted R^2	.076**	.049*	.073**	.062**	.223***	.194***	.169***	.161***	.019	.065*	.136***	.060*	.032*	.022	.036*	.071**
H-channel						-.148		.173								
S-channel										.186						
V-channel												-.142				
Symmetry left/right	.257									-.199		-.158				
Symmetry up/down													-.145			
Edge density					**.42**	**-.413**	**.367**	**.376**	.173	**.203**						
Self-similarity											**-.310**	**.228**				
Fourier slope		-.171														**-.232**
Fourier sigma	**-.231**	**.196**	**-.228**	**-.200**	**-.208**	**.319**					**-.318**	.132				
1st-order entropy			.144	.152							.126		-.217	.231		
2nd-order entropy					**.364**	**-.253**	**.307**	**.271**				-.146	**.303**	**-.293**	**-.204**	**.275**
Variance Pa						**-.200**										
Variance Pf	**.275**				.199		**.346**	**.345**		**-.224**	**-.217**					

Notes. Significant at * $p < .05$, ** $p < .01$ and *** $p < .001$
